# Spherical Polybutylene Terephthalate (PBT)—Polycarbonate (PC) Blend Particles by Mechanical Alloying and Thermal Rounding

**DOI:** 10.3390/polym10121373

**Published:** 2018-12-11

**Authors:** Maximilian A. Dechet, Juan S. Gómez Bonilla, Lydia Lanzl, Dietmar Drummer, Andreas Bück, Jochen Schmidt, Wolfgang Peukert

**Affiliations:** 1 Institute of Particle Technology, Friedrich-Alexander-Universität Erlangen-Nürnberg, Cauerstraße 4, D-91058 Erlangen, Germany; maximilian.dechet@fau.de (M.A.D.); juan.s.gomez@fau.de (J.S.G.B.); andreas.bueck@fau.de (A.B.); jochen.schmidt@fau.de (J.S.); 2 Interdisciplinary Center for Functional Particle Systems, Friedrich-Alexander-Universität Erlangen-Nürnberg, Haberstraße 9a, D-91058 Erlangen, Germany; 3 Collaborative Research Center 814—Additive Manufacturing, Am Weichselgarten 9, D-91058 Erlangen, Germany; lanzl@lkt.uni-erlangen.de (L.L.); drummer@lkt.uni-erlangen.de (D.D.); 4 Institute of Polymer Technology, Friedrich-Alexander-Universität Erlangen-Nürnberg, Am Weichselgarten 9, D-91058 Erlangen, Germany

**Keywords:** polybutylene terephthalate (PBT), polycarbonate (PC), polymer blend particles, spheroidization, additive manufacturing, Raman microscopy, SEM staining, trans-esterification

## Abstract

In this study, the feasibility of co-grinding and the subsequent thermal rounding to produce spherical polymer blend particles for selective laser sintering (SLS) is demonstrated for polybutylene terephthalate (PBT) and polycarbonate (PC). The polymers are jointly comminuted in a planetary ball mill, and the obtained product particles are rounded in a heated downer reactor. The size distribution of PBT–PC composite particles is characterized with laser diffraction particle sizing, while the shape and morphology are investigated via scanning electron microscopy (SEM). A thorough investigation and characterization of the polymer intermixing in single particles is achieved via staining techniques and Raman microscopy. Furthermore, polarized light microscopy on thin film cuts enables the visualization of polymer mixing inside the particles. Trans-esterification between PBT and PC during the process steps is investigated via vibrational spectroscopy and differential scanning calorimetry (DSC). In this way, a new process route for the production of novel polymer blend particle systems for SLS is developed and carefully analyzed.

## 1. Introduction

Polymer-based additive manufacturing (AM) techniques allow for the building of highly customized parts that are not accessible with conventional subtractive manufacturing [1]. Technologies like fused filament fabrication (FFF), stereolithography (SLA), and binder jetting offer the manufacturing of a wide variety of rigid to flexible parts. Most of these parts, however, do not provide the toughness and stability needed for parts subjected to mechanical loads [2]. Selective laser sintering (SLS), a technology employing polymer powders, which are applied layer-by-layer and selectively sintered with a laser, yields dense parts with a high mechanical strength [3]. Because of the high demands of the process on the powder material properties, especially regarding the size distribution, shape, and thermal properties (i.e., processing window [4], melt viscosities [5], and isothermal crystallization [6]), the choice of commercially available SLS powders is quite limited [7]. The most frequently used material is polyamide 12 (PA12), but polyamide 11 (PA11), polyamide 6 (PA6), polystyrene (PS), poly(methyl methacrylate) (PMMA), polypropylene (PP), thermoplastic elastomers (TPE), high-density polyethylene (HDPE), and polyaryletherketones (PAEKs) are also available, but not yet optimized for SLS processing [2]. Several approaches to provide SLS one-component powders from other polymers are reported in the literature (e.g., a process chain [8] employing wet grinding of polymers [9] followed by rounding the comminution product in a heated downer reactor [10], a melt emulsification process [11], spray agglomeration [12], spray drying [13], and precipitation-based processes [14,15]).

However, processes to fabricate polymer blends in powder form are still rare. A route to introduce novel material properties to the SLS process is the combination of available powders to manufacture blend polymer parts [16], as follows: via physical mixtures of powders, Salmoria et al. processed blends of PA12/HDPE [17,18], PA6/PA12 [19]; PMMA/PS [20]; and, recently, polybutylene terephthalate(PBT)/PA12 [21] in a SLS machine. The processing of a physical mixture of PBT and PC (polycarbonate) has been reported recently by Greiner et al. [22]. However, reports on blend particles with the intra-particle mixing of polymers, which would allow for much smaller polymer domains, are scarce. Blend particles of PA12/PEEK, PA12/PP, and PA12/PC have been manufactured by cryogenic comminution and mechanical alloying [23,24]. During this process, which is commonly conducted in a high-energy ball mill, the polymer feed materials are repeatedly deformed, comminuted, and fused, resulting in mixed blend particles [25,26].

The comminution of polymers often yields powders made up of irregular shaped particles [9,27] with a low bulk density and unfavorable flowability, resulting in poor SLS processability. The improvement of the flowability and packing density of the comminution product can be achieved by a shape modification, namely, the thermal rounding in a downer reactor [28]. This has been successfully demonstrated for, for example, the rounding of grinded particles of polystyrene (PS) [28], polybutylene terephthalate (PBT) [8], or PBT/glass composite particles [29]. Blends of PBT–PC may provide excellent mechanical properties paired with high chemical resistance, rendering it highly interesting for many demanding applications (e.g., in automotive, as custom-made fuel manifolds in racing cars). The semi-crystalline polymer (PBT) provides high thermal and chemical resistance, while the amorphous (PC) contributes high impact resistance, toughness, and improved heat deflection behavior [30,31]. The SLS processing of PBT–PC blends is challenging, as this system does not exhibit a process window according to the theory of isothermal laser sintering [32]. No gap between the onset of the melting peak and the crystallization peak exists, as PBT is semi-crystalline and PC is amorphous [22]. By using blends with a semi-crystalline and an amorphous component, shrinkage during cooling could be reduced, and the enhanced part properties with a higher dimensional accuracy can be achieved.

For the SLS process, the intra-particle mixing of the two phases is beneficial, as the mixing of PBT and PC within a single particle (e.g., size of around 60 µm) would avoid parts with clearly separated phases, and enhance the benefits of PBT–PC blends, like the ability of part functionalization (e.g., metallization [33]). By utilizing a PBT–PC blend powder for selective laser sintering, the advantages of powder-based additive manufacturing (e.g., the fabrication of complex geometries without the need for any tools or molds) are accessible with a material that shows a higher toughness and increased impact resistance as compared to the standard laser sintering material, PA12. Furthermore, this blend system is of scientific interest, as it is composed of two intrinsically immiscible polymers [34], which become miscible [35] upon the formation of a co-polymer generated by trans-esterification [36], which acts as a compatibilizer [37]. The trans-esterification can be catalysed by the addition of, for example, an alkyl titanium compound [38] or magnesium oxide (MgO) [31]. However, for manufacturing purposes, the trans-esterification reaction is often deliberately inhibited [30] by the addition of phosphite compounds [39], as higher degrees of trans-esterification impair the blend properties [40]. Therefore, a PBT–PC blend powder without pronounced trans-esterification is desired for selective laser sintering.

In this study, by combining the co-grinding [41] of PBT and PC in a planetary ball mill typically used for mechanical alloying [42,43], and the subsequent thermal rounding, spherical PBT–PC blend particles are made readily available. MgO was added in some co-grinding experiments to study whether trans-esterification may be induced mechano-chemically during milling [44], or whether thermal trans-esterification during rounding occurs. The manufactured particles were investigated via vibrational spectroscopy (FTIR; Raman), to identify the trans-esterification products, as well as to assess the mixing of both polymers in a single particle. Polarized light microscopy and scanning electron microscopy (SEM) investigations on ruthenium tetroxide (RuO_4_) stained samples [39] allow for the direct observation of the different polymers in particles. Furthermore, DSC experiments were conducted in order to investigate whether trans-esterification happens during SLS process-like conditions.

## 2. Materials and Methods

### 2.1. Materials

Injection-grade polybutylene terephthalate (PBT Ultradur B 4520, BASF SE, Ludwigshafen, Germany) granules, and injection-grade bisphenol-A-based amorphous polycarbonate (PC Makrolon 2405, Covestro AG, Leverkusen, Germany) granules were pre-comminuted separately in a rotary impact mill (Pulverisette 14, Fritsch GmbH, Idar-Oberstein, Germany) equipped with a 0.5 mm sieve ring operated with a pin rotor speed of 20,000 rpm. Liquid nitrogen was used for cooling to embrittle the feed material. The obtained comminution products (x_50,3_ < 0.3 mm) were used as feed materials for planetary ball milling. Magnesia (MgO Luvomag M 072, Lehmann&Voss&Co. KG, Hamburg, Germany) was used in some experiments as a trans-esterification catalyst [31].

### 2.2. Experimental

#### 2.2.1. Co-Grinding in a Planetary Ball Mill

The comminution and simultaneous intermixing behavior of the PBT and PC feed powders was achieved by planetary ball milling (Pulverisette 7 classic line, Fritsch GmbH, Idar-Oberstein, Germany). Planetary ball mills provide a high energy input and are frequently applied for similar tasks with metals, like mechanical alloying [42,45]. The following experimental conditions were applied: Grinding vessels of 12 mL volume made of Yttria-stabilized zirconia (YTZ) were equipped with 50 grinding balls (YTZ) of 5 mm size. Two vessels were used in parallel and were filled with 2 g of each polymer, PBT and PC. Co-grinding was performed in the sealed vessels at an ambient atmosphere at a rotational speed of the sun wheel of 600 rpm. For each full revolution of the sun wheel, the grinding vessels (i.e., the planet wheel) counter-rotated twice, leading to high stress energies. After each consecutive 20 min of grinding, the mill was stopped for 2 min to allow the system to cool down, in order to avoid excessive thermal stressing of the polymers. Moreover, the direction of the rotation was changed after each cooling period. Besides the experiments with a (pure) PBT–PC mixture, grinding experiments under the addition of a 0.1 wt % MgO trans-esterification catalyst were also performed.

A basic estimation of the maximum stressing energy, defined as the kinetic energy *E_kin_* of a grinding ball traversing the full inner diameter of the grinding chamber, is given by the literature [46], employing Equation (1).

(1) $$E_{kin}=m_{GB}*\alpha *d_{GC}\;\left(\alpha =R\omega^{2}\right)$$

The kinetic energy is given by the mass of a single grinding bead, *m_GB_*; the acceleration of a grinding bead, *α;* and the inner diameter of the grinding chamber, *d_GC_*. The acceleration, *α*, is calculated using the effective radius of the revolution, *R*, which is 70 mm for the used mill, and the angular velocity, ω, to the power of 2. Using the parameters described above, the maximum stress energy, *E_kin_*, of a single grinding bead is around 11.8 mJ, however, the effective stress energy a particle experiences typically will be much lower (see e.g., [46,47]). The temperature inside of the grinding vessel was measured every 20 min cycle for 240 min (i.e., 12 grinding cycles); after 60 min, the temperature remained at 40 °C for the rest of the experiment. Although the effective local temperature at the moment of stressing is hardly accessible experimentally, under the chosen conditions, the conversion to the co-polymer by thermal trans-esterification is assessed to be negligible. The observed temperature increase of the stressed sample is reasonable for the chosen stressing conditions [44].

#### 2.2.2. Thermal Rounding

**Figure 1:**
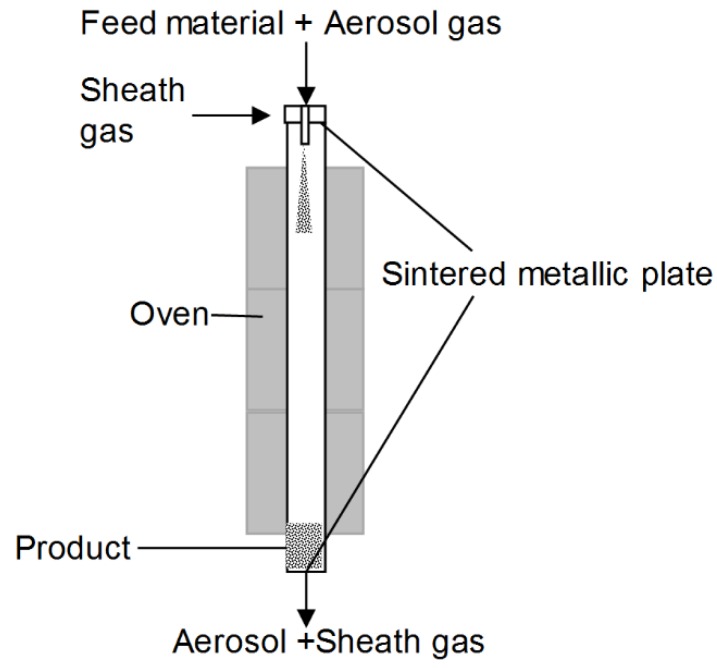
Schematic representation of the heated downer reactor.

The downer setup is made up of a heated stainless-steel pipe, an aerosol generator unit, and a separation unit to recover the rounded particles. A scheme of the setup is shown in Figure 1. The comminuted particles are dispersed in nitrogen (5.0, Linde plc, Dublin, Ireland) by means of a brush disperser unit (RGB 100, Palas GmbH, Karlsruhe, Germany) at a gas flow rate of 2.49 m^3^/h and a pressure of 1 bar. The mass flow of the solid was chosen to be 34 g/h under these conditions. The aerosol is centrally fed into the head of the reactor. A nitrogen sheath gas flow is employed to reduce the interactions of the particles and the wall. The volumetric flow of the sheath gas is controlled by a mass flow controller (EL-Flow, Wagner Mess-und Regeltechnik GmbH, Offenbach am Main, Germany). The sheath gas is homogeneously distributed over the downer cross section surrounding the aerosol gas by means of a sintered metal plate (SIKA-R20, GKN Sinter Metals GmbH, Radevormwald, Germany). The reactor has a diameter of 100 mm and a length of 6 m. The heating of the reactor is realized by a three-stage oven (Thermal Technology GmbH, Bayreuth, Germany) with a height of 4.5 m. The temperature of the upper section (5.5–4 m) is set above the melting point of PBT. In this section, the gas and the particles are gradually heated from an ambient temperature to the melting point of the polymer. The temperature in the second section (4–2.5 m) is set to the melting point of the polymer, and allows for the complete melting of the particles. Driven by surface tension (c.f. minimization of surface free energy, and, thus, surface area), the molten particles progressively acquire a spherical shape. In the third heating section (2.5–1 m), the temperature is set below the melting point; the melt droplets are allowed to solidify. The last cooling step is achieved at the lower unheated end of the reactor (1–0 m), where the obtained spherical particles are separated from the gas flow by means of a sintered metallic plate (SIKA-R20, GKN Sinter Metals GmbH, Radevormwald, Germany). A more detailed description of the geometry of the downer reactor has been reported previously [48].

The temperature of the three heating sections of the oven was set to 280, 230, and 150 °C, respectively. In order to investigate the influence of the volumetric flow rate of sheath gas on the rounding process, it was set to 3 and 7 m^3^/h.

For a sheath gas flow of 3 m^3^/h, the temperature achieved in the center of the reactor cross section in the second heating section was very close to the melting temperature of PBT (T_m,PBT_ = 227–230 °C) [8], indicating a homogenous radial and axial temperature profile. In this section, all of the particles are supposed to be in the molten state. For a volumetric flow of sheath gas of 7 m^3^/h, the temperature in the center of the cross section of the reactor is below the melting temperature of PBT, however, the melting point temperature of PBT is achieved in the near wall region; the solid is concentrated in the near wall region (r/D >0.85, with D being the inner diameter of the downer reactor and r being the radial position) [49,50]. Thus, it is expected that the rounding of some particles occurs there. In the third heating section, where the temperature is set below the melting point of PBT and in the final unheated section of the downer, the radial temperatures profiles are reversed; the temperature in the center of the reactor is higher than in the near-wall region. In these last two regions, the particles solidify, respectively, and are in the solid state.

Because of the complex radial temperature and solid concentrations profiles in the downer, the determination of the (real) residence time distribution of the particles above the melting point is not straightforward. To come up with a characteristic residence time, *τ*, the following simplifications have been made: (i) the particles and the gas phase have the same velocity, and (ii) plug flow and (iii) a homogenous radial and axial temperature distribution apply. Moreover, if it is supposed that the polymer particles only get rounded in the second downer section, which is heated to the melting temperature, the residence time of particles above the melting point can be calculated as follows:

(2) $$\tau =\frac{\pi D_{d}^{2}L_{m}\rho_{tmelt}}{4{\dot{V}}_{0,total}\rho_{0}}$$

In Equation (2), D is the inner diameter of the downer, *L_m_* is the length of the second (middle) heating section, and ${\dot{V}}_{0,total}$ is the total volume flow resulting from the contribution of the aerosol and sheath gases at room temperature. $\rho_{0}$ and $\rho_{tmelt}$ are the density of nitrogen at room temperature and at the melting temperature of the polymer, respectively. The calculated average residence time of the particles above the melting point are 4.52 s and 2.61 s for a flow rate of 3 and 7 m^3^/h, respectively.

### 2.3. Characterization

#### 2.3.1. Laser Diffraction Particle Sizing

The particle size distributions were determined by laser diffraction particle sizing using a Mastersizer 2000 (Malvern Panalytical GmbH, Kassel, Germany) equipped with a wet dispersing unit Hydro 2000. Before measurement, the powders were pre-dispersed in water with the addition of sodium dodecyl sulfate (SDS, Merck, KGaA, Darmstadt, Germany) and ethanol (96%, denatured) in an ultrasonic bath for a few minutes. During the measurements, a stirring rate of 3500 rpm and ultrasonication at 100% power were set at the Hydro 2000 unit.

#### 2.3.2. Scanning Electron Microscopy

The particle shape and surface morphology were characterized by scanning electron microscopy (SEM) using a Gemini Ultra 55 (Carl Zeiss AG, Oberkochen, Germany) operated at an acceleration voltage of 1.0 kV. A SE2 detector and a through-the-hole detector were used for the unstained and stained samples, respectively. The rounded polymer blend particles were stained with in-situ formed RuO_4_ as described by Brown [51] and Trent [52], in order to visualize the distribution of PBT and PC.

#### 2.3.3. Raman Microscopy

The spectra were collected with a LabRAM HR Evolution Raman microscope (Horiba, Kyoto, Japan) equipped with a frequency-doubled Nd-YAG laser (λ = 532 nm; grating 1800 g/mm; 50X large working distance (LWD) objective, NA = 0.5; Hole = 1000 µm; Slit = 1000 µm) for Raman shifts between 100 cm^−1^ and 3200 cm^−1^, employing an integration time of 30 s. The spectral resolution was 0.5 cm^−1^. Based on Abbe’s law (λ/NA), the spatial resolution of this setup can be estimated to be 1.06 µm.

#### 2.3.4. Polarized Light Microscopy

The inner microstructure of PBT–PC particles was analyzed by polarized light microscopy on thin sections of 10 µm thickness. The particles were embedded into epoxy resin and thin sections were cut using a microtome. The morphology of the particles was analyzed with an Axioplan microscope (Carl Zeiss AG, Oberkochen, Germany).

#### 2.3.5. Infrared Spectroscopy

The infrared (IR) spectra were recorded in attenuated total reflection (ATR) in the spectral range of 4000 cm^−1^ to 400 cm^−1^, at a resolution of 2 cm^−1^, using an FTS 3100 FTIR spectrometer (Agilent Technologies Inc., formerly Varian, Santa Clara, CA, USA). The reported spectra were ATR corrected and were normalized to their respective maximum absorbance; the baseline correction was done manually.

#### 2.3.6. Differential Scanning Calorimetry

The thermal analysis of the polymers by differential scanning calorimetry (DSC) was performed with a DSC8000 (Perkin Elmer Inc., Waltham, MA, USA). To approximate the thermal profile that the polymer powder experiences during the laser sintering (SLS) process, the following temperature program was applied: First, the sample (typical mass: 7–9 mg) was heated to 185 °C with 20 K/min, and then to 237 °C with 10 K/min; then, an isothermal step was applied for a duration of 1 min. This temperature profile simulates the melting of the deposited powder in the process chamber during laser exposure. Then, the polymer melt was cooled to 215 °C at 20 K/min, which is the building chamber temperature range one would choose for the polymer [8,22]. The system was held at 215 °C for various times for 30 min before finally being cooled to room temperature with 10 K/min. While the applied heating and cooling rates are not comparable to the very fast rates caused by direct illumination with the laser in SLS [53], the set temperature profiles shall mimic process conditions (e.g., building chamber temperature).

To characterize the thermal material behavior after solidification, a second heating cycle was performed. There, the system again was heated to 185 °C with 20 K/min, and then to 237 °C with 10 K/min, and was cooled to room temperature at 10 K/min. This temperature profile was chosen to assess the thermal properties of the blend material after the simulation of a SLS-like temperature profile and to investigate prospective trans-esterification during selective laser sintering. Trans-esterification occurring during the first heating run would be characterized by a decrease in the crystallinity of the blend, and, thus, a less pronounced melting endotherm [54].

A second temperature profile was applied to induce trans-esterification. There, the system was heated to 185 °C with 20 K/min and then to 237 °C with 10 K/min, and was held there for 45 min before being cooled to 185 °C with 20 K/min and to room temperature with 10 K/min. The second heating was analogous to the one described above for the first temperature profile.

## 3. Results and Discussion

### 3.1. Co-Grinding and Rounding of PBT and PC

**Figure 2:**
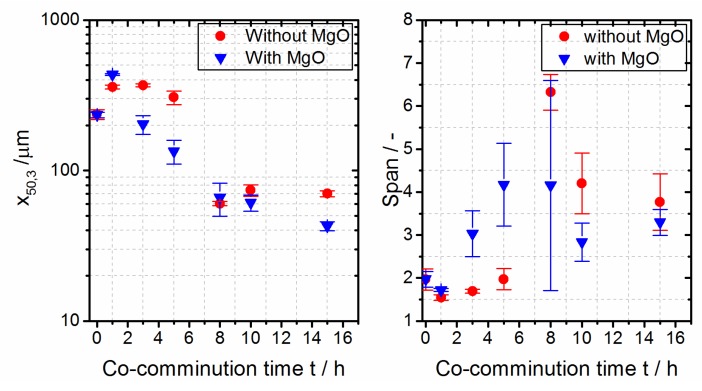
Dependence of the particle size x_50,3_ and span on process time during co-comminution of a polybutylene terephthalate (PBT) and polycarbonate (PC) mixture (1:1 (wt./wt.); feed mass load 2 g; with/without MgO catalyst). **Left**: Volume average mean particle size x_50,3_. **Right**: Span, (x_90,3_ − x_10,3_)/x_50,3_ as a measure of the broadness of the particle size distribution. For experimental conditions, see Section 2.2.1.

The temporal evolution of the volume averaged mean product particle size, x_50,3_, during the co-comminution of a PBT–PC mixture with and without the addition of a MgO trans-esterification catalyst is illustrated in Figure 2 (left). For the stressing conditions, please refer to Section 2.2.1. The feed material consisted of pre-comminuted PBT and PC particles with size distributions characterized by x_10,3_ = 79 µm, x_50,3_ = 239 µm, and x_90,3_ = 550 µm, and x_10,3_ = 91 µm, x_50,3_ = 194 µm, and x_90,3_ = 363 µm for PBT and PC, respectively. For the stressed 1:1 PBT:PC mixture without MgO (c.f. circles), an increase of the mean particle size after approximately 1 h was observed because of mechanical deformation, that is, the change of shape (c.f. flattening; Figure 5 (right)) of the particles [55] because of the acting compression and shear stresses in the planetary ball mill [56,57]. In the further course of comminution, the product particle size gradually decreased down to a product particle size of x_50,3_ in the range of 60 to 70 µm for process times of more than 8 h. The addition of 0.1 wt % of MgO accelerated the co-comminution considerably. Without the addition of MgO, there was no significant reduction of the volume averaged mean particle size from 1 to 5 h; with the addition of MgO, the product particle size x_50,3_ decreased considerably during the same time. After 8 h of co-comminution, particle sizes of about 60 to 70 µm were obtained. In contrast to the grinding experiment with the PBT–PC mixture without the MgO addition, in the experiment under the addition of the trans-esterification catalyst, a further reduction of the product particle size was observed (c.f. x_50,3_ = 42.6 µm for 15 h), indicating that the oxide influences the breakage mechanism of the polymer. The reason for this behavior so far has not yet been fully clarified. Possible explanations could be mechanochemical effects [58] or an increasing brittleness caused by the incorporation of hard MgO particles into the polymer matrix. The increased brittleness changes the fracture behavior by lowering the impact strength. Such effects have been described for some polypropylene-based particulate composites in the literature [59]. For a PBT-glass composite powder, similar observations were made during wet grinding [29].

**Figure 3:**
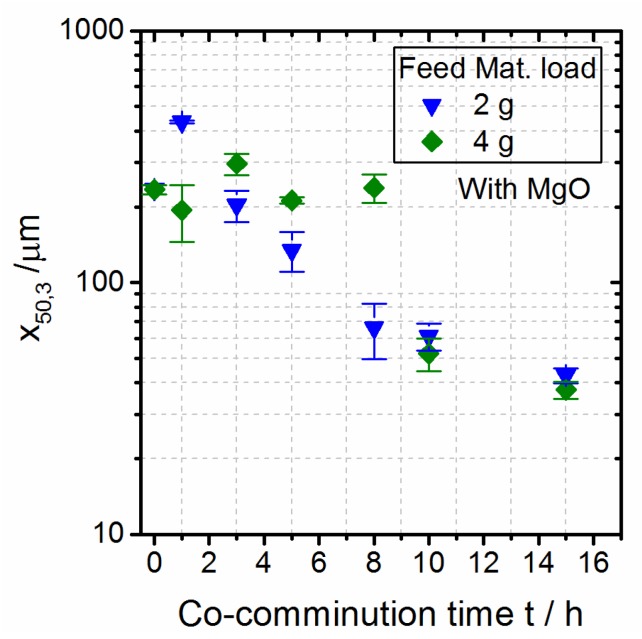
Dependence of the particle size x_50,3_ on process time during co-comminution of a PBT–PC mixture (1:1 (wt./wt.); with MgO catalyst): influence of feed load.

Figure 2 (right) illustrates the span (defined as (x_90,3_ − x_10,3_)/x_50,3_) as a function of the co-comminution time, as follows: It first increases with the increasing comminution time, reaching a maximum at around 8 h, and then decreases in the further course of comminution, indicating a narrowing of the particle size distribution of the product. After 15 h of comminution, spans around 3.3 to 3.8, typical for products obtained by planetary ball milling [9], are found. Figure 3 shows the effect of the polymer feed load on the temporal evolution of the particle size. As expected, a slower reduction of x_50,3_ over time is observed with an increase in the feed load [46,47,60]. For prolonged grinding (c.f. data for 10 h and 15 h), similar product particle sizes x_50.3_ were obtained independent of the chosen feed load, although a smaller span was observed at a higher feed load.

**Figure 4:**
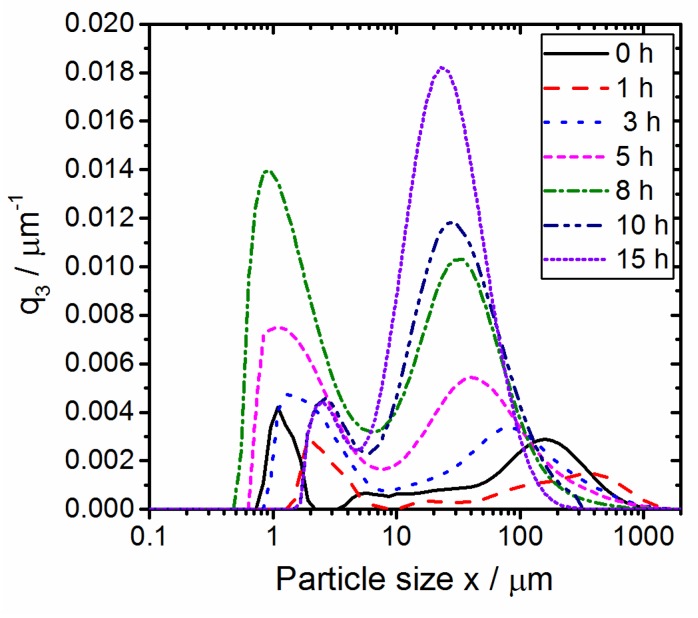
Particle size distributions (q_3_, volume density) of co-comminuted PBT–PC mixture (1:1 (wt./wt.); feed mass load 2 g; with MgO catalyst) in dependence on the process time. For stressing conditions, please refer to Section 2.2.1.

**Figure 5:**
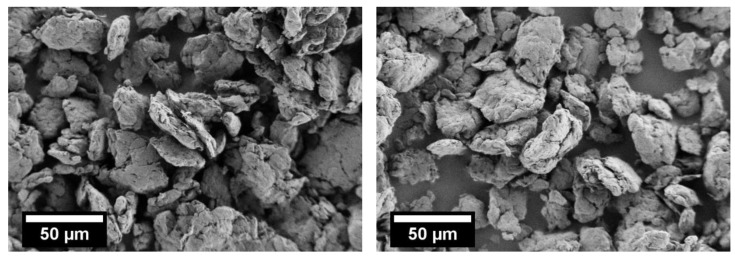
SEM image of PBT–PC blend particles obtained by planetary ball milling for 10 h (**left**) and 15 h (**right**), (600 rpm; 5 mm grinding beads).

In Figure 4, the volume-based particle size distributions, q_3_, of the co-comminuted PBT–PC powders (with MgO addition) in dependence on the comminution time are depicted. Over grinding time the products are characterized by the evolution of a bimodal size distribution, with one fraction between 0.8 µm and 3 μm and another fraction between 20 and 400 μm, depending on the comminution time. The broadness and position of the modes of the distributions change as the comminution time increases. As outlined above, after a grinding time of 1 h, the particle size distribution determined via laser diffraction is shifted towards larger values as compared to the feed material, which most likely is a measurement artefact. The plastic deformation of the feed results in flattened particles (c.f. Figure 5); as the size measurement is done in a flow cell, the prolate particles orient in the flow direction [61], the light scattered by these oriented particles is measured by the ring detector, and the size is overestimated [55,62]. For co-comminution times larger than 1h, the particle size distribution shifts progressively to smaller values. Moreover, the width of the fractions decreases with the increasing comminution time.

The obtained co-comminuted PBT–PC product is characterized by irregular shaped particles, as exemplified in Figure 5, for the materials obtained after 10 and 15 h of processing. The general morphology indicates that the polymeric material is jointly kneaded, causing overlapping structures of PBT and PC with many distinct boundaries. Polymer powders obtained by comminution are typically are characterized by a low flowability and packing density, leading to problems in processing in the SLS machines, which may cause components with disadvantageous mechanical properties [32,63]. One approach to improve the SLS processability of comminuted polymer powders is thermal rounding [8,28,64], which will be discussed in the following for the co-comminuted PBT–PC material. The rounding experiments were performed for the comminution products obtained after 10 h and 15 h, respectively.

### 3.2. Rounding

**Figure 6:**
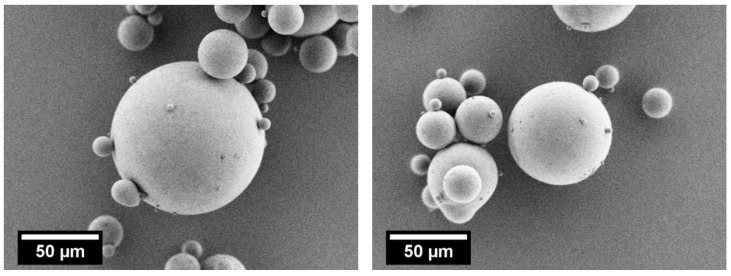
SEM images of thermally rounded PBT–PC blend particles. **Left**: Grinding time 10 h and sheath gas flow rate 3 m^3^/h. **Right**: Grinding time 15 h and sheath gas flow rate 3 m^3^/h.

Figure 6 depicts the SEM images of PBT–PC particles obtained by thermal rounding of the comminution product shown in Figure 5 at a flow rate of sheath gas of 3 m^3^/h. As discussed in Section 2.2.2, also for conditions where the melting point of the polymer is not reached in the center of the downer cross section, a rounded product is obtained because of the high solid concentration typically found in the near wall region. Consequently, independent of the sheath gas flow rate, the particles are predominantly spherical and are characterized by smooth surfaces (Figure 6). Powders with these characteristics typically show higher packing densities and better flowability than comminution products. Schmidt et al. [8] demonstrated in a comparable approach, that the thermal rounding of wet comminuted PBT powders allows for an increase in the bulk density and an improvement of powder flowability, as determined by the powder tensile strength measured with a modified Zimmermann tensile strength tester [65,66].

**Figure 7:**
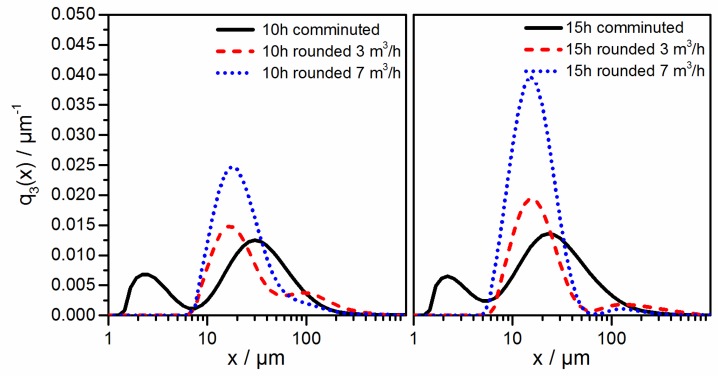
Particle size distributions of comminuted feed material and PBT–PC blend particles obtained by thermal rounding.

The effect of the rounding process on the product particle size distributions is depicted in Figure 7. As compared to the size distribution of the comminution product used as the feed material (c.f. black line), the mode at 3 µm disappears and the second mode shifts towards lower values (approximately 20 µm). Moreover, a third mode appears at approximately 150 µm, because of agglomeration; the fines fraction (<10 µm) disappears completely. A further investigation of this effect and its control is currently being performed and will be reported on in subsequent contributions. The influence of the sheath gas flow rate is also depicted in Figure 7, namely: at 7 m^3^/h (dotted line), the agglomeration of particles is reduced as compared to 3 m^3^/h (dashed line). This is due to the lower interactions between the polymer particles (c.f. lower solids concentration at 7 m^3^/h and, thus, smaller collision kernel), and the lower residence time of the particles in the downer at the higher sheath gas flow rate.

The reduction of the agglomeration at a higher gas flow rate is also expressed in Table 1, as follows: the x_50,3_ of the product obtained at 7 m^3^/h gas flow as measured by laser diffraction particle sizing was even lower than the median particle size of the not rounded powders, which most likely is due to the shape change (c.f. Figure 5) induced by rounding. Additionally, the high deviation of the ideal spherical shape, considered in the scattering model to determine the particle size distribution, may lead to artifacts in the PSDs of the flake-like comminuted particles determined by laser diffraction, resulting in an overestimation of the particle size and broader modal peaks [55,67,68,69]. After rounding, these artifacts are eliminated, resulting in narrower peaks and a decline of the fine fraction.

### 3.3. Vibrational Spectroscopy

**Figure 8:**
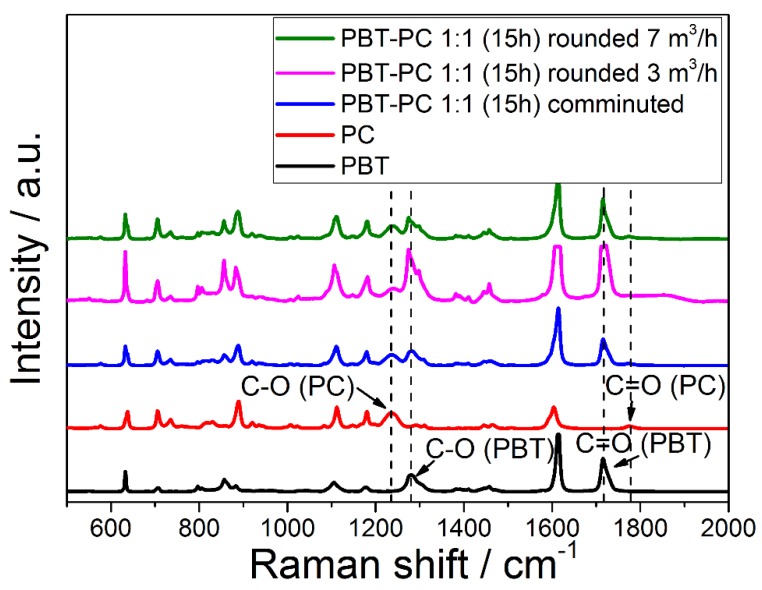
Raman spectra of feed polymers, the co-comminuted PBT–PC mixture and rounded PBT–PC blend particles. Dashed lines mark C–O and C=O bands of PBT and PC, respectively. For assignment of the spectra, refer to the legend.

For the characterization of the processed polymers, PBT and PC, vibrational spectroscopy (IR; Raman) is employed. Although both polymers show similar bands because of similar functionalities in their molecular structure, the C–O and the C=O stretching bands in the Raman spectrum are at specific locations, and thus allow for the identification of PBT and PC; the C=O stretching band of PBT is found at 1720 cm^−1^, and for PC, the respective band is located at 1775 cm^−1^. C–O stretching bands are found at 1280 cm^−1^ and 1235 cm^−1^ for PBT and PC, respectively [70]. In order to investigate the composition of the PBT–PC particles obtained after thermal rounding, Raman microscopy was employed (for instrumental details, see Section 2.3.3.). Figure 8 depicts the Raman spectra of the PBT and PC particles obtained by comminution and rounded PBT–PC blend particles. In the spectra of the co-comminuted PBT–PC mixture and in the spectra of the rounded PBT–PC blend particles, the C–O and C=O stretching bands (ν), being typical for both of the polymers, are observed; ν_C=O_ of PC (at 1775 cm^−1^) is intrinsically weak [71], while ν_C-O_ (at 1775 cm^−1^) of PC is clearly found in the spectra of the co-comminution product and the rounded product. The bands at 1720 cm^−1^ and 1280 cm^−1^ can be assigned to the ν_-C=O_ and the ν_-C-O_ of PBT. During the comminution process, fragments of the two polymers are kneaded into each other and are thereby jointly mixed (c.f. Figure 5). During the rounding process, obviously, no complete de-mixing occurs, as the particles showing the spectral features of both of the polymers are obtained. The Raman analysis gives the spectral information of the confocal detection volume in the single particles. This detection volume will be significantly larger than the spatial resolution of the optical setup, as the selected hole size (1000 µm) and laser intensity, necessary in order to detect sufficient Raman signal, result in wider confocal detection volumes [72]. A detailed view on the blend distribution structure on the surface is achieved via SEM on stained samples (c.f. Section 3.4), while the analysis in the particle volume is achieved by polarized light microscopy on thin sections (c.f. Section 3.5). The presence of appreciable amounts of trans-esterification products in the co-comminuted or the rounded particles could not be confirmed. An indicator for (pronounced) trans-esterification is a strong fluorescence, which easily leads to detector saturation [23]. During the recording of the Raman spectra, no remarkable fluorescence was observed. No differences between the samples with and without the addition of MgO were observable in the spectra.

**Figure 9:**
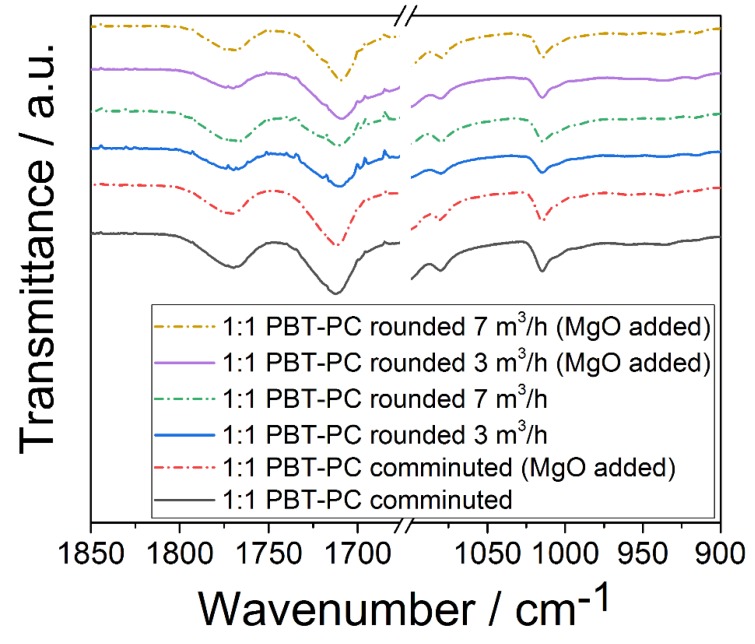
IR spectra of comminuted and rounded PBT–PC powders (with and without MgO addition during processing). All of the samples were comminuted for 15 h. For assignment of the spectra, refer to the legend.

A direct observation of the potentially formed co-polymers by trans-esterification is possible via IR spectroscopy for concentrations higher than 3.5 mol %, whereas lower concentrations are only indirectly detectable by changes in the PBT crystallinity [37]. Devaux et al. [40] identified several ester and carbonate groups, which formed during the trans-esterification reaction of PBT and PC and could assign specific IR absorption bands, namely: aromatic esters (1070 cm^−1^ and 1740 cm^−1^) and an aromatic carbonate (1780 cm^−1^). Figure 9 depicts the IR spectra of the co-comminuted PBT–PC mixture and the rounded PBT–PC blend particles (with and without addition of MgO). The PBT specific C=O stretching at around 1718 cm^−1^ [73] and the PC specific C=O stretching, asymmetrical O–C–O stretching, and symmetrical O–C–O stretching at 1770 cm^−1^, 1080 cm^−1^, and 1015 cm^−1^, respectively [74], are well observable. However, the IR spectra show no signs of the reported bands being specific for trans-esterification products, which indicates no significant trans-esterification of PBT and PC during processing.

### 3.4. Staining/SEM Imaging

**Figure 10:**
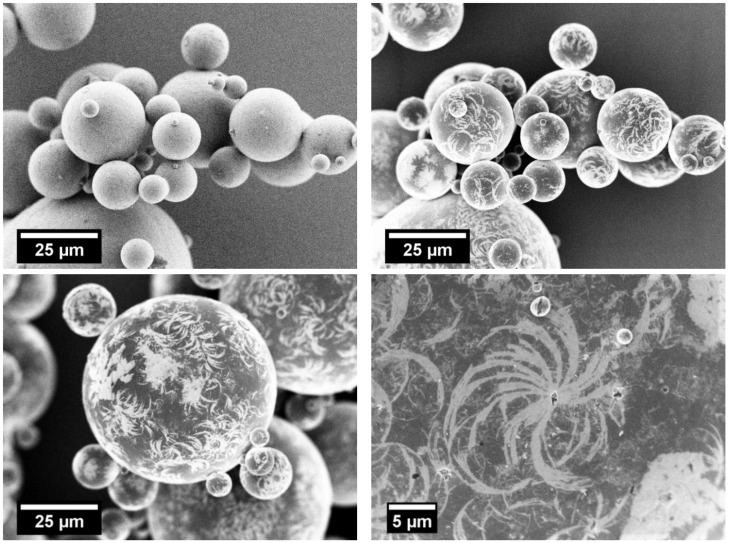
**Top**: SEM images of stained spherical PBT–PC particles (15 h; 3 m^3^/h) recorded using a SE2-detector (left, top) and a through-the-hole detector (right, top; right and left, bottom). **Bottom** (left): Stained spherical PBT–PC particles (15 h; 3 m^3^/h). **Bottom** (right): Magnification of crescent shaped PC domains on particle surface (10 h; 3 m^3^/h).

The staining of the rounded PBT–PC blend particles with in-situ formed ruthenium tetroxide (RuO_4_) and the subsequent SEM imaging allows for the discrimination of both polymers because of the material contrast (observable with the through-the-hole-detector) caused by the differences in the diffusion rate of the RuO_4_ into the polymers. In the preliminary staining experiments with comminuted PC and rounded PBT particles, the proper preparation conditions were identified and an assignment of the polymers with respect to their characteristic material contrast during SEM imaging was established, as follows: stained PC appears bright, indicating a higher ruthenium concentration per volume as compared to PBT, which appears darker (lower ruthenium concentration). The SEM images of the stained rounded blend particles (3 m^3^/h) are given in Figure 10—the top images (SE2 (left) vs. through-the-hole detector (right)) confirm that by means of the through-the-hole detector, the regions made up of PC and PBT can be discriminated. These experiments confirm that blend particles made up of both polymers can be produced by our approach (Figure 10, top (right)). Moreover, detailed images (Figure 10, bottom) show that some larger areas are made up of PC, and many small PC areas are found at the particle surface. The SEM imaging of the stained rounded particle systems leads to the conclusion that we were able to manufacture blended spherical PBT–PC particles by applying a process route consisting of dry co-grinding and thermal rounding in a downer reactor.

### 3.5. Polarized Light Microscopy

**Figure 11:**
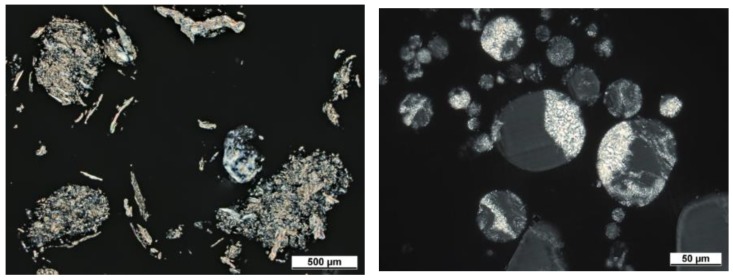
Morphology of PBT–PC particles after comminution (**left**) and rounding (**right**) investigated by polarized light microscopy of thin sections.

While SEM imaging and staining is appropriate for investigating the phase distribution on the surface of the individual polymer particles, it is also necessary to analyze the morphology within the particle. Figure 11, depicts the thin sections observed under polarized light. The semi-crystalline PBT can be assigned to the regions with a higher refractivity, which appear as bright regions under polarized light. The amorphous PC appears as dark grey and can be well differentiated from the black epoxy background. Therefore, the mixing of both components within grinded and spheroidized particles is observable. Figure 11 (left) depicts porous structures (c.f. black background is visible within the structure) made up of filamentary, comminuted particles, which are formed by the breakage and subsequent kneading of the fragments attributable to the shearing during comminution. Such morphologies are known for the mechanical alloying of ductile–ductile systems [25,75]. In this sample, PBT and PC can be hardly distinguished. In contrary to the comminuted particles, for the rounded blend particles (Figure 11 (right)), the phases of the two components become obvious. The PBT, appearing bright because of a higher refractivity of its spherulites, and the PC are well dispersed within one particle. However, some de-mixing and consolidation of the polymer phases in the particles, compared to the comminuted samples, occurs during thermal rounding. This is most likely caused by the viscous flow of the polymer during the rounding and the limited miscibility of PBT and PC, especially for very low concentrations of the trans-esterified co-polymer.

**Figure 12:**
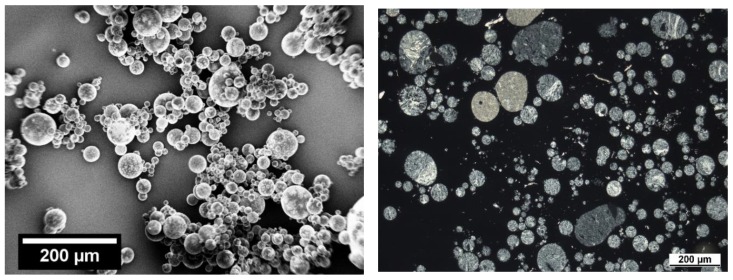
Comparison of spherical blend particles (15 h; 3 m^3^/h) analyzed via SEM (stained samples, **left**) and polarized light microscopy (**right**).

Taking into account all of the applied characterization techniques, a thorough view on the resulting blend structure after mechanical alloying and thermal rounding can be achieved. While the FTIR spectroscopy shows that both polymers are present in the bulk, the Raman microscopy proves the presence of polymer blend particles (i.e., PBT and PC are present in a single particle). These results are complemented by the SEM analyses of the stained samples and polarized light microscopy, namely: these methods allow for the direct observation of the polymer distribution in the particle. In Figure 12, an overview is given for the stained particles via SEM (left) and thin sections via polarized light microscopy. While both techniques show that both of the polymers are well mixed in each particle, a homogenous or structured distribution cannot be identified.

### 3.6. Thermal Characterization

Dynamic scanning calorimetry (DSC) was performed to investigate whether and to which extend a trans-esterification of PBT and PC occurs in the melt phase [34] at temperature profiles mimicking the laser sintering process. There, the laser-exposed polymer remains in a molten state and will only slowly crystallize [6]. The second heating in the DSC was used to analyze the effects of the simulated laser sintering temperature profile (see Section 2.3.6).

**Figure 13:**
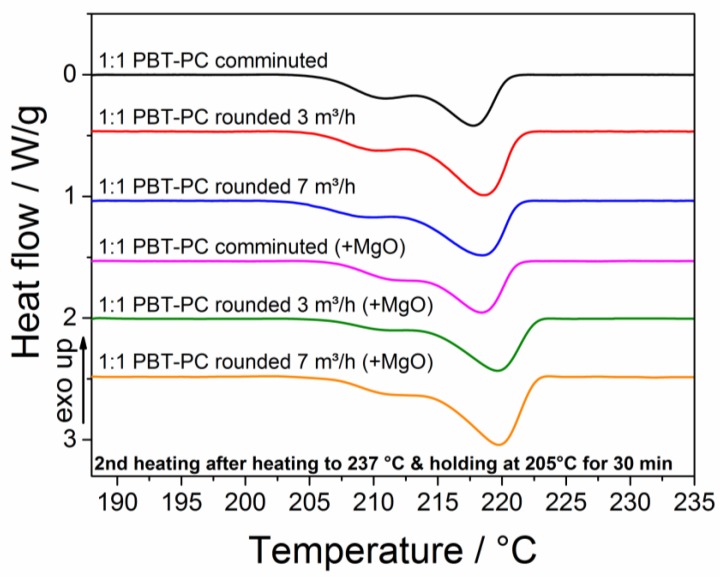
Thermograms (second heating) of comminuted and rounded PBT–PC particles with and without the addition of MgO after simulated laser sintering temperature profile (see Section 2.3.6).

Figure 13 summarizes the thermograms for the second heating for the comminuted and rounded particles, with and without the addition of MgO during processing. In all of the thermograms, a melting endotherm with a peak maximum at 221 °C is found, and no differences in the position of the aforementioned peak temperature is observed for the different samples. Obviously, the relatively fast recrystallization of PBT at the building chamber temperature prevents the remarkable conversion of PBT and PC to the trans-esterified product; isothermal crystallization half-times in the range of 4 to 5 min have been reported for PBT at 205 °C [76,77], and 10 to 15 min for the temperature range 210 °C to 215 °C [78]. The trans-esterification reaction kinetics, especially at relatively low temperatures close to the melting point, are reported to be much slower [79]. Consequently, even for rather long process times (of hours), being typical in SLS formation, remarkable amounts of trans-esterified polymer do not occur, which is advantageous with respect to the mechanical properties of the produced parts. Upon increasing the trans-esterification, blend properties like the chemical resistance and heat deflection temperature decline. Consequently, for the manufacturing of the functional parts needed to perform under demanding environments, trans-esterification is unfavorable [30,80]. However, if trans-esterification is favored, the building temperature should be set as high as possible, as higher temperatures impede the isothermal crystallization kinetics and accelerate the trans-esterification kinetics.

**Figure 14:**
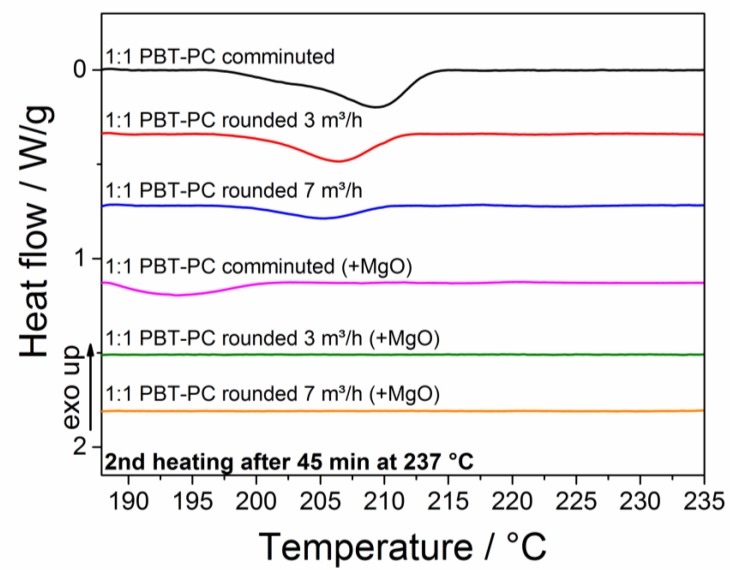
Thermograms (second heating) of comminuted and rounded PBT–PC particles with and without the addition of MgO after melt annealing for 45 min at 237 °C.

Further insights into the trans-esterification of the PBT–PC particles were achieved by the deliberate induction of the trans-esterification reaction with annealing above the PBT melting temperature. Figure 14 depicts the thermograms (second heating) of comminuted and rounded PBT–PC particles with and without the addition of MgO after annealing at 237 °C for 45 min. The increasing conversion of the two polymers PC and PBT (i.e., the trans-esterification and the catalyzing effect of MgO on the conversion rate) is visible. The thermogram of the comminuted sample shows a flat endothermic peak with the peak maximum shifting from 221 °C (simulated sintering process, c.f. Figure 14 and Section 2.3.6 for temperature profile) to around 210 °C (melt annealing, 237 °C). The rounded samples show larger temperature shifts of the melt endotherm peak temperature to 207 °C and 204 °C (melt annealing) for the sheath gas flow rates during rounding of 3 m^3^/h and 7 m^3^/h, respectively. The sample comminuted under MgO addition shows a very flat peak in the thermogram in the range of 195 °C, confirming that the catalyzing effect of the MgO c.f. the melt endotherm decreases with an increase in the trans-esterification product being present. The thermograms of the rounded samples showing no melting endotherm confirm that the product is completely amorphous; the formation of the co-polymer inhibits the crystallization of PBT [81]. A decrease of PBT crystallinity, indicated by a flatter melt endotherm, and a shift of the melt endotherm towards lower temperatures with increasing trans-esterification, has been discussed by Tattum et al. [36]. The degree of PBT-crystallinity for the samples can be calculated by relating the PBT-specific enthalpy of fusion to the heat of fusion of a perfect PBT crystal (145.5 J/g [82]). The calculated PBT crystallinities for the different samples treated with the simulated SLS temperature profile and melt annealing are listed in Table 2. The samples treated with the simulated SLS temperature profile show crystallinities Χ_PBT,SLS profile_ in the range of 23% to 29%. Comparable crystallinities in the range of 32% to 36% for the same PBT grade have been reported for wet grinded and rounded PBT samples [8]. The melt annealed samples show significantly lower degrees of crystallinity Χ_PBT,melt anneal_ in the range of 13% to 0%, which is a clear indicator of increasing trans-esterification.

The observed larger shifts in the position of the melt endotherm temperature found for the rounded samples in comparison to the comminuted samples can be explained by the differences in the particle/aggregate structure, namely: the comminuted particles appear rough and show a morphology that indicates kneading of the different polymers into single (aggregate) particles. Those aggregate particles show some porosity and voids (c.f. Figure 11 (left), which leads to loose contact between the polymer–polymer boundaries in the aggregate particle. Therefore, the trans-esterification reaction occurring at the polymer–polymer boundary might be hindered. These boundaries change during the thermal rounding process, as the melting period allows for a viscous flow of the polymers, leading to closer contact of the polymer–polymer boundaries (c.f. Figure 11 (right)).

In summary, even though the employed temperature profile (c.f. Section 2.3.6) is far from the real process, like the heating and cooling rates introduced by the laser [53], remarkable trans-esterification during the “typical” temperature time profiles and laser exposure strategies one would apply in selective laser sintering seems unlikely. According to Devaux et al. [79], the degree of trans-esterification of a 50:50 PBT–PC without the addition of a catalyst held for 45 min at a temperature of 237 °C is comparable to a sample of the same composition held at 224.5 °C for around 100 min. Hopfe et al. [35] quantified the formed co-polymer via NMR for a 1:1 PBT–PC sample held at 260 °C, and measured concentrations of 1.5 mol %, 2.7 mol %, 6.0 mol %, and 12.1 mol %, for 7 min, 13 min, 22 min, and 45 min at this temperature, respectively. From this, even longer times for the induction of significant trans-esterification are needed for temperatures in the range of the building chamber temperature for the system considered. However, this could allow for the manufacturing of functionally graded structures, as the degree of trans-esterification of the polymers and, thus, to a certain extent, the mechanical properties of the specific areas of a device built from the powder could be tailored by involved exposure strategies (e.g., based on a simultaneous laser beam melting approach [83]). Areas in the powder bed, where (high) trans-esterification is favored, are selectively and are repeatedly exposed with high energy deposition and fast scanning rates, whereas areas where no co-polymer is wanted are treated according to the “typical” SLS exposure strategy. This could result in parts with locally tailored properties and functionalities.

## 4. Conclusions

In this account, an approach to obtain non-trans-esterified spherical PBT–PC blend particles by a two-stage process was outlined; co-grinding allowed for a size reduction of the feed polymers and intermixing of the obtained fragments, while rounding of the grinding product in the heated downer reactor enabled shape control. The obtained spherical particles are of a size distribution being well suited for SLS processing. The intermixing of PBT and PC during co-grinding could be proven via the Raman microscopy of single particles. No significant trans-esterification, because of neither mechanochemistry during grinding nor thermal activation during rounding, could be observed by IR spectroscopy. Deeper insights into the inner blend particle microstructure were gained by the polarized light microscopy of thin sections. These findings are complementary to the SEM observations of stained spherical particles, where the identification of PBT and PC domains in single particles was possible. The behavior of the blend particles during SLS process-like thermal conditions was investigated via DSC. No significant trans-esterification should be expected during SLS processing, even if MgO is added as a catalyst. Only for longer time periods above the PBT–PC melting point, could such reactions be observed, giving the opportunity to tailor the degree of trans-esterification of the built device by specific exposure strategies. In order to determine the material behavior of the blend particles during SLS, and to assess the correlations between the polymer distribution in the particles and in the manufactured parts thereof, a thorough part characterization employing the techniques discussed here will be performed in the future.

## Figures and Tables

**Table 1:** Comparison of product particle size x_50,3_ of blend particles obtained at different stressing and rounding conditions.

Milling Time/h	V_sheath_/m^3^/h	x_50,3_/µm	Span/-
10	not rounded	56.5	4.20
10	3	92.1	2.73
10	7	34.4	4.73
15	not rounded	51.5	3.76
15	3	126.2	3.54
15	7	21.5	6.29

**Table 2:** Calculated polybutylene terephthalate (PBT) crystallinities. PC—polycarbonate.

Sample	Χ_PBT,SLS profile_/%	Χ_PBT,melt anneal_/%
1:1 PBT–PC comminuted	23	13
1:1 PBT–PC rounded 3 m^3^/h	28	8
1:1 PBT–PC rounded 7 m^3^/h	26	4
1:1 PBT–PC comminuted (+MgO)	20	4
1:1 PBT–PC rounded 3 m^3^/h (+MgO)	26	0
1:1 PBT–PC rounded 7 m^3^/h (+MgO)	29	0
